# Carcinosarcoma of the Gallbladder with Chondrosarcomatous Differentiation and Intracytoplasmic Eosinophilic Hyaline Globules (Thanatosomes): A Report of a Case and Review of the Literature

**DOI:** 10.1155/2019/9697235

**Published:** 2019-02-06

**Authors:** Jumana A. Alratroot, Amani A. Joudeh, Samir S. Amr

**Affiliations:** ^1^Department of Pathology, Faculty of Medicine, Imam Abdulrahman Bin Faisal University, Dammam, Saudi Arabia; ^2^Department of Pathology and Laboratory Medicine, King Fahad Specialist Hospital, Amer Bin Thabet Street, Dammam 31444, Saudi Arabia

## Abstract

A 52-year-old woman presented with abdominal pain and vomiting. Computed tomography (CT) scan of the abdomen revealed a huge exophytic gallbladder mass displacing or invading the surrounding structures. The patient underwent radical cholecystectomy, transverse colectomy, distal gastrectomy, and liver bed resection. Histologically, the tumor showed both carcinomatous and sarcomatous components, with prominent chondrosarcomatous differentiation. In addition, several malignant cells showed intracytoplasmic eosinophilic hyaline globules (Thanatosomes). The tumor showed metastatic deposits to the omentum, the liver, and the peripancreatic lymph nodes. We report this unusual case and present a review of all cases of carcinosarcoma of the gallbladder with chondrosarcomatous differentiation.

## 1. Introduction

Carcinosarcoma of the gallbladder, also referred to by some authors as malignant mixed tumors and sarcomatoid carcinoma, is a rare malignant neoplasm representing less than 1% of all gallbladder malignant tumors [1]. This highly malignant tumor is composed of a carcinomatous component, which can be glandular or squamous or both, intermingled with sarcomatous elements which can include undifferentiated spindly homologous component, or heterologous component including osteosarcomatous, chondrosarcomatous, or rhabdomyosarcomatous differentiation. The presence of heterologous elements differentiates carcinosarcomas from spindle and giant cell undifferentiated carcinomas [1]. These tumors are highly aggressive and behave like a carcinoma. In a review of 3038 patients with gallbladder cancer collected by the Surveillance, Epidemiology, and End Results (SEER) Program, 11 patients with carcinosarcoma were identified and 9 of them died as a result of the tumor [2].

Herein, we report a 52-year-old woman with carcinosarcoma of the gallbladder featuring an anaplastic sarcomatous component associated with prominent chondrosarcomatous differentiation and extensive metastases. In addition, sarcomatous tumor cells contained hyaline eosinophilic globules, a novel finding in these tumors. We present a review of all cases of carcinosarcoma of the gallbladder with chondrosarcomatous differentiation.

## 2. Case Report

A 52-year-old Saudi Arab woman presented to the Emergency Department of a local hospital complaining of right upper quadrant pain accompanied by vomiting of 5 day-duration. The patient had a progressive right upper quadrant abdominal pain for the last five months that increased in severity with fatty meals radiating to the back. She had no history of jaundice, change of urine color, or weight loss. On physical examination, there was a palpable abdominal mass in the right upper quadrant that is 8 cm below the costal margin. Abdominal ultrasound showed the gallbladder to have thick wall with large irregular shaped soft tissue mass arising from the fundus, measuring 8 x 6 cm. This was seen infiltrating the surrounding sub-hepatic fat planes and inseparable from the transverse colon. There were no focal hepatic lesions; dilated intrahepatic biliary radicals or significant enlarged lymph nodes were noted.

**Figure 1:**
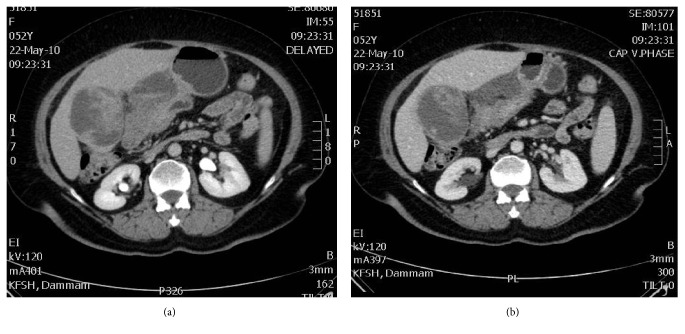
(a) and (b) Abdominal CT scan demonstrating a large exophytic mass within the gallbladder.

The patient was referred to our hospital for evaluation and management. Laboratory work-up revealed normal hematological parameters. Liver function tests revealed an elevated GGT (65 unit/liter), low albumin (32 g/L) and unremarkable total bilirubin, alkaline phosphatase, ALT, and AST. Renal function tests were within normal limits. Serum carcinoembryonic antigen (CEA) and *α*-fetoprotein (AFP) were normal, but carbohydrate antigen 19-9 (CA 19-9) was elevated (154.33 IU/ml). CT scan of the abdomen showed a gallbladder mass with huge exophytic component, displacing the surrounding structures with few small portal lymph nodes (Figure 1). No hepatic lesions were identified and a few small bilateral pulmonary nodules of uncertain significance were noted. The patient underwent laparotomy with radical cholecystectomy, transverse colectomy, distal gastrectomy, omentectomy, and liver bed resection.

## 3. Pathological Findings

The gross pathological specimen consisted of portion of liver with attached gallbladder, portion of stomach, segment of large bowel and attached omentum. The gallbladder measured 13.0 cm in length and 6.0 cm in diameter. On opening the gallbladder, a large polypoid tumor mass was seen filling the lumen measuring 11.0 cm in length and 6.0 cm in diameter. The tip of the mass was friable reddish brown in color. The root which was mainly present in the body is soft reddish tan in color. No gallstones were identified in the lumen of the gallbladder. On cut section, fleshy tumor was seen arising within the gallbladder extending to surrounding tissue, forming rubbery soft tan and hemorrhagic nodules the largest measured 11.0 cm in diameter, reaching to the wall of the stomach and colon surrounded by omental tissue. A large nodule measuring 8.0 cm beneath the omentum adherent to the liver was noted. Another hemorrhagic nodule measuring 7.8 cm was present between the liver and the stomach. On opening the stomach and the large bowel, the mucosal linings of both were unremarkable.

**Figure 2:**
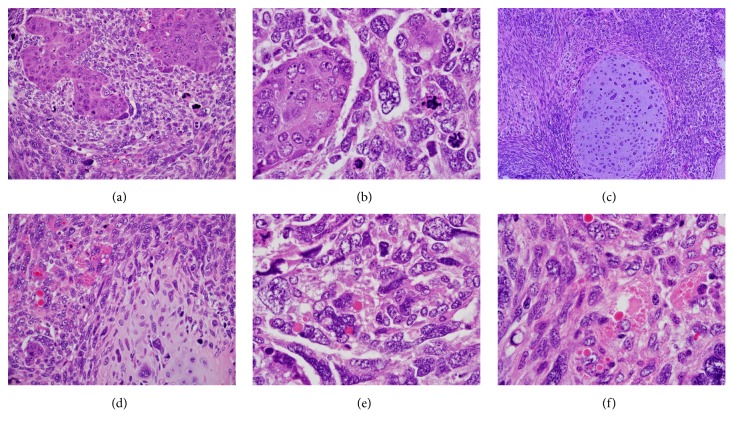
(a) Solid nests of malignant epithelial cells surrounded by malignant mesenchymal cells with pleomorphic nuclei and mitotic figures. H&E x200. (b) Solid nest of malignant epithelial cells with squamoid features surrounded by pleomorphic sarcomatous cells with bizarre nuclei. Note the presence of three atypical mitotic figures. H&Ex400. (c) An island of malignant cartilage with malignant chondrocytes featuring pleomorphic large nuclei, surrounded by spindly sarcomatous cells. H&E x200. (d) Island of malignant cartilage with adjacent pleomorphic sarcomatous cells containing eosinophilic hyaline globules 9Thanatosomes). H&E x200. (e) Intracytoplasmic eosinophilic hyaline globules (Thanatosomes) within the cytoplasm of sarcomatous cells. H&E x400. (f) Numerous eosinophilic hyaline globules (Thanatosomes), some are extruded into adjacent stroma. H&E x400.

Histological examination of the intraluminal large polyp of the gallbladder showed a biphasic highly malignant tumor featuring both solid epithelial nests within highly malignant sarcomatous stroma (Figures 2(a) and 2(b)) with scattered islands of malignant cartilage seen (Figure 2(c)). The tumor cells had hyperchromatic pleomorphic nuclei with many huge monstrous bizarre multilobulated forms noted (Figure 2(d)). Many mitoses, mostly atypical (tripolar), were seen. In addition, several scattered tumor cells showed the presence of intracytoplasmic eosinophilic hyaline globules (EHGs) that were variable in size from finely small granules to large globules (Figures 2(e) and 2(f)). Some EHGs were seen in adjacent connective tissue stroma to disrupted or degenerated cells that contained these globules. The wall was heavily infiltrated by the tumor in the area that the polyp had arisen from. Sections from the gallbladder away from the tumor showed evidence of xanthogranulomatous cholecystitis.

Infiltrating highly sarcomatous component was seen in the pericolic fat, perigastric fat, hepatic parenchyma, and perihepatic fibrous tissue. Foci of malignant solid epithelial nests and malignant glandular formation surrounded by sarcomatous stroma with scattered islands of malignant cartilage were identified in the omental mass. Peripancreatic lymph nodes revealed metastatic deposits of sheets and clusters of malignant cells with epithelioid features in half of the lymph nodes which were identified.

**Figure 3:**
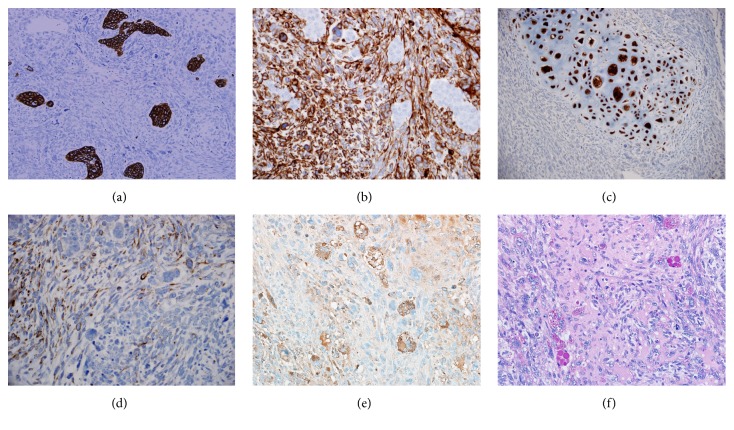
(a) Cytokeratin AE1/AE3 immunostain featuring positive nests of malignant epithelial cells and negative staining of sarcomatous component. X100. (b) Vimentin stain featuring positive staining of sarcomatous cells, and negative staining of epithelial nests. (c) Island of malignant cartilage staining positively for S100-protein X100. (d) Desmin stain featuring scattered positive mesenchymal sarcomatous cells. X200. (e) Alpha 1-antitrypsin positive staining of eosinophilic hyaline globules. X100. (f) Eosinophilic hyaline globules (EHGs) stain positive for PAS stain with diastase. X100.

Immunohistochemical stains revealed the malignant epithelial cells to be positive for cytokeratin (Figure 3(a)) and negative for vimentin. On the other hand, the sarcomatous cells were positive for vimentin (Figure 3(b)) and negative for cytokeratin. The malignant cartilage stained positive for S-100 protein (Figure 3(c)), and the spindly mesenchymal cells were focally positive for desmin (Figure 3(d)). The eosinophilic hyaline globules were negative for CAM5.2 (CK8 and CK18) and for alpha fetoprotein (AFP). They were positive for alpha 1-antitrypsin (Figure 3(e)) and alpha 1-antichymotrypsin. EHGs were positive for PAS stain with and without diastase (Figure 3(f))

In view of these findings, the diagnosis of gallbladder carcinosarcoma with anaplastic sarcomatous and chondrosarcomatous components was confirmed. This neoplasm was staged as stage III (pT4 pN2 pMx).

The postoperative course was uneventful. The patient was referred to the Medical Oncology team 7 weeks following the surgical procedure. A management plan was set and included six cycles of adjuvant chemotherapy (gemcitabine 1000 mg/m^2^ and cisplatin 25 mg/m^2^). Upon the patient's request, she continued her chemotherapy treatment near her home and no further follow-up information was obtained afterwards.

## 4. Discussion

Carcinosarcomas of the gallbladder (CSGB) are quite rare. Okabayashi et al. in a review of surgical outcome of 131 cases of carcinosarcoma of the hepatobiliary tract reported that, between 1970 and 2012, there were 59 cases CSGB documented in the world literature [3]. Zhang et al. in 2008 undertook a computerized search in PubMed of the United States National Library of Medicine and ISI Web of Science database through the Internet and found 68 cases. Their search included cases labelled as carcinosarcoma, sarcomatoid carcinoma, and spindle cell carcinoma of the gallbladder [4]. They selected only cases with a reliable histopathological diagnosis of CSGB. Since then, additional several cases had been reported. 

There had been some controversy regarding the pathogenesis of this tumor, reflected by the multitude of names designated for it, including malignant mixed tumor [5–7], sarcomatoid carcinoma [8–11], spindle cell carcinoma [12, 13], and malignant mixed mesodermal tumor [14]. Five theories were postulated to explain the histological mixture of epithelial and mesenchymal malignant elements in CSGB. One theory proposed that the mesenchymal tissue represented a reactive process; a second theory considered the mesenchymal tissue as a true sarcoma (the collision tumor or convergence multiclonal theory); a third theory hypothesized that the tumor is a carcinoma with metaplastic changes to sarcoma (the divergent monoclonal theory); a fourth theory pointed to embryonic rest origins; and the last theory was the totipotential stem cell hypothesis [15, 16].

To add to this controversy regarding its pathogenesis, CSGB had been categorized as a “true” carcinosarcoma if the mesenchymal elements showed differentiation into neoplastic bone, cartilage or skeletal muscle [1, 40], or as “so-called” carcinosarcoma if the malignant mesenchymal component did not show differentiation, but exhibited spindly or pleomorphic cells without differentiation into malignant bone or cartilage or rhabdomyoblastic cells [8, 12]. Adenocarcinoma is almost always a constant finding in the carcinomatous component of CSGB, but in one third there are foci of keratinizing squamous cell carcinoma [1]. In a review of 68 cases, Zhang et al. demonstrated that adenocarcinoma was the most common epithelial component, representing 79.2% of the cases. A mixture of adenocarcinoma and squamous cell carcinoma was observed in 11.3%. 9.4% of the cases demonstrated the presence of squamous cell carcinoma only [4]. The sarcomatous component could be homologous featuring malignant spindly mesenchymal cells with fibrosarcoma-like features, or heterologous featuring malignant stromal cells admixed with heterologous elements such as malignant osteoid, malignant cartilage or rhabdomyoblasts. The homologous spindle cell component was observed in 44.6% of the 68 cases reviewed by Zhang et al., while osteoid formation was the least observed (5.4%) in that study. The cases reported by Aldovani et al. and Mehrotra et al. showed exclusively malignant osseous tissue in various stages of differentiation [14, 40]. Ishihara et al. documented a case of CSGB that showed rhabdomyosarcomatous elements, confirmed by immunohistochemistry and electron microscopy, as the only heterologous component [41]. Wang et al. reported a case of CSGB accompanied with bile duct tumor thrombi that showed rhabdomyosarcomatous differentiation as the sole heterologous component of the sarcomatous elements of the tumor [16]. A mixture of more than one heterologous element such as malignant osteoid and cartilage were observed in several cases [4]. Zhang et al. divided 45 cases of CSGB they retrieved from the literature that had details of their histological findings. They divided the cases into five groups by their mesenchymal component type, including spindle cell, chondroid, rhabdomyomatous, osteoid, and admixture of two or more component types. They found on statistical analysis no significant difference in the outcome of patients with various histological types, thus suggesting that mesenchymal component of CSGB has little prognostic value [4].

We collected all cases of CSGB that exhibited chondrosarcomatous differentiation (Table 1). There were 30 cases, including the current one. The cases were collected from the world literature over 93 years dating back to 1925 [4–7, 13, 17–39]. There were 8 males and 21 females, with one case without available data on age and sex of the patient [19]. Male to female ratio was 1:2.6. Their ages ranged from 38 to 91 years (Mean age 67 years). In addition to the presence of chondroid differentiation, twelve tumors showed additional osteoid differentiation [5, 7, 17, 21, 22, 25, 28, 31, 33, 36, 38, 39], two cases showed additional rhabdomyosarcomatous differentiation only [7, 26], and one case showed additional both rhabdomyosarcomatous and osteoid differentiation [24]. Metastatic spread, particularly to the liver was documented in 18 cases (62%). Data related to survival, excluding autopsy cases, were available on 20 patients. Two patients died within days following surgery due to massive pulmonary embolism [7], or cardiogenic shock [32]. Twelve patients died of metastatic spread of the tumor or sepsis within a period of time ranging from 1 month to 17 months (mean 4.9 months), six of them within three months. Six patients were alive at the time of last follow up, with their survival ranging from 3 to 54 months (mean 33.8 months). Allover survival was 13.5 months, ranging from 0 to 54 months. Prolonged survival had been emphasized in two patients, one survived 48 months [31], and the other 54 months [29].

In a review of 59 cases of CSGB that underwent surgical resection, there were 18 males and 39 females, with male to female ratio of 1:2.2. Two-thirds of the patients (38 patients) were 65 years or older. The survival rate by the end of one year was 42.8%, and the 5-year survival rate was 37.6%. The median survival in months was 7.0 ± 2.0 [3]. Another review of 36 patients of CSGB, who underwent surgical management, showed median survival to be 7.0 months with a range from 4.4 to 9.6 months. There were 10 males and 26 females with male to female ratio of 1:2.6, with a mean age of 67.7 years [42]. A meta-analysis study of 68 cases of CSGB showed the mean age of the patients was 68.8 years. There were 16 males and 52 females with male to female ratio of 1:3.25. The mean survival was 17.5 months, with a median of 5 months, and a range between 0 and 85 months [4].

Comparing the data from these studies to ours, the dominance of CSGB in the females is apparent. The mean age is very close in all four studies, ranging from 65 to 68.8 years. Survival data were similar There are some variations in the mean of survival in months following surgical intervention, but is not greatly significant. One of the interesting points made in the meta-analysis study was correlation of the race of the patients with survival. They found that Japanese patients (27 cases) had longer survival time than non-Japanese (24 cases), with mean survival of 19.9 months for the Japanese vs 11.5 months for the non-Japanese, with a median of 6 vs 4 months. In our review, 11 out of 29 patients (38%) were Japanese, with average survival of 16.9 months, compared with nine non-Japanese, with available data on their survival, who had average survival of 6.2 months. Three of the Japanese patients had prolonged survival of 24, 48, and 54 months, respectively (Table 1). Japanese patients with carcinoma of the gallbladder had better survival when compared with other high incidence countries such as Chile and India. Multiple factors might result in such better prognosis in Japanese, including genetic variations, early detection, and better surgical techniques [4].

Abdominal mass, fever, nausea, vomiting, abdominal pain, anorexia, loss of weight, and painless jaundice are some of the nonspecific presentations of this tumor [24, 31]. Our patient was admitted initially to the Emergency Department due to abdominal pain with vomiting but without jaundice. An abdominal mass was palpated and was confirmed by ultrasound examination. CSGB are usually polypoid and their size varied from 3 to 15 cm. and can fill the lumen of the gallbladder [1]. Our patient had a tumor that filled the lumen measuring 11 cm in maximum dimension. In a review of 41 patients with CSGB who had data on tumor size and survival, it was demonstrated that patients with tumors measuring ≥ 5 cm had a significantly shorter survival when compared with patients who had tumors measuring less than 5 cm. [4].

Gallstones were observed in 8 out of 9 cases (88%) of CSGB in one review [24], in 14 out of 18 cases (77%) in another review [23], and in 66.7% of cases in a third review [4]. This high association between gallstones and CSGB is similar to what is observed with carcinoma of the gallbladder. Our patient did not have gallstones found in the lumen of the gallbladder but got xanthogranulomatous cholecystitis.

Historically, the concept of “carcinosarcoma” was introduced by Rudolf Virchow in 1865 [43]. Karl Landsteiner, an Austrian pathologist and the discoverer of the ABO blood groups, was credited with the description of the first case of CSGB in 1907 in a pathology museum specimen [44]. Saphir and Vass made in 1938 a review of all cases of carcinosarcoma in various organs, dating back to 1878 [45]. They collected 153 cases from 25 organs, with most frequent cases encountered in uterus, breast, lung, larynx, esophagus, thyroid, and urinary bladder. There were only two cases of CSGB, including Landsteiner's case. They were of the opinion that majority of these tumors are of carcinomatous origin, and their critical review of the documented cases indicated that their carcinosarcomatous nature was questionable. It is interesting to note that these authors made their observations on routinely H&E stained histological sections fifty years before the introduction of immunohistochemical stains as a diagnostic tool that demonstrated the positive staining of the spindly “sarcomatous” for cytokeratin and other epithelial markers confirming their nature as epithelial in origin.

To the best of our knowledge, none of the cases of CSGB on record had demonstrated the presence of eosinophilic hyaline globules (EHG). The present case showed the presence of several EHG within the cytoplasm of the mesenchymal sarcomatous spindly and pleomorphic cells, as well as free within the surrounding extracellular tissue. They were variable in size and were highlighted by Periodic Acid Schiff (PAS) stain. Such EHG had been labelled as “Thanatosomes,” a term proposed by Papadimitriou et al. denoting a degenerative phenomenon related to apoptosis, common to all cell types, benign or malignant, resulting from cytoplasmic blebbing and nuclear fragmentation followed by phagocytosis by neighboring cells or auto-phagocytosis, accompanied by increased membrane permeability to plasma proteins [46]. In their study, they examined 80 tumors from various organs that contained hyaline globules by light and electron microscopy and did immunohistochemical studies on them. Most cells containing EHG stained with immunohistochemical stains for all plasma proteins examined alpha-1-antitrypsin, ferritin, C3, kappa and lambda light chains, and IgG, indicating increased plasma membrane permeability. Among those 80 tumors, there were two cases of hepatocellular carcinoma but no cases of gallbladder cancer.

In another study related to Thanatosomes in gastrointestinal epithelium, Dikov et al. examined 2230 biopsies of normal and pathological epithelium. EHGs were very rarely found in normal epithelium (1.1%), but their number increased significantly in specimens with ischemic injury (47%) and benign regenerative proliferation (70%). They concluded that Thanatosomes represented a relatively constant and useful histologic marker of enhanced cell turnover and ischemic injury [47]. Thanatosomes had been dubbed as “Death Bodies” in a report on 2 cases of malignant phylloides tumor, both of which showed numerous Thanatosomes, to the point of dominating the histological appearance and masking the stromal element [48]. Our case is the first CSGB to feature this peculiar finding. 

## Figures and Tables

**Table 1:** Cases of Carcinosarcoma of Gallbladder with Chondrosarcomatous Differentiation (1925-2018).

No	Author Country Year (Reference)	Sex	Age	Microscopic findings	IHC carcinoma	IHC sarcoma	Metastasis and Outcome
1	Kritsch Germany 1925 [17]	F	59	Carcinoma and sarcoma containing cartilage and bone.	Not done	Not done	Autopsy diagnosis.
2	Billi Italy 1964 [18]	F	59	Carcinoma with myxochondrosarcoma.	Not done	Not done	Alive four months after surgery.
3.	Edmondson USA 1967 [19]	?	?	Squamous cell carcinoma with foci of adenocarcinoma. Undifferentiated sarcoma with islands of malignant cartilage.	Not done	Not done	One of two cases of CSGB reported in AFIP fascicle on tumors of gallbladder without clinical data.
4	Sagi and Gyori Hungary 1972 [20]	F	79	Adenocarcinoma and fibro-chondromyxosarcoma.	Not done	Not done	Autopsy diagnosis
5	Higgs et al. USA 1973 [5]	M	77	Neoplastic glandular structures. Mucinocarminophilia was demonstrated. Neoplastic mesenchymal spindle-shaped cells, malignant cartilaginous and metaplastic osseous tissue.	Not done	Not done	Patient had tumor spread within bile duct. He developed infection of biliary tract by Klebsiella, and died on 29th postoperative day. Metastatic spread to liver and bile ducts with fibrosarcoma pattern predominated over all other neoplastic elements.
6	Mansori and Cho USA 1980 [6]	M	81	Most of the tumor is composed of bizarre pleomorphic cells. Foci of malignant gland formation. Spindly sarcomatous component with areas of malignant cartilage.	Not done	Not done	Tumor invaded adjoining liver tissue. Patient died within four weeks postoperatively. He had leukocytosis, suspected for sepsis and was placed on chemotherapeutic agents with no effect.
7	Von Kuster and Cohen USA 1982 Case 1 [7]	F	91	Well to poorly differentiated adenocarcinoma. Cellular stroma with spindle cells in herringbone pattern. Foci of malignant cartilage One focus of malignant osteoid.	Not done	Not done	Exploratory laparotomy showed a. mass in the gallbladder, with extensive metastases to the liver. Patient died on the third post-operative day of massive pulmonary embolism. Autopsy showed metastases to liver, left adrenal gland and para-aortic lymph nodes.
8	Von Kuster and Cohen USA 1982 Case 2 [7]	F	77	Moderately to poorly differentiated adenocarcinoma, contained mucin. Fibrosarcomatous stroma with small focus of malignant cartilage, and rhabdomyoblasts.	Not done	Not done	Cholecystectomy was done. No evidence of spread of the tumor. No chemotherapy was given. Patient was alive 31 months following surgery.
9	Hasegawa et al. Japan 1983 [21]	F	73	Solid nests of carcinoma with signet ring cells. Extensive areas of chondrosarcoma. Osteoid formation is present.	Not done	Not done	Patient had also adenocarcinoma of sigmoid colon. Patient had metastases to the liver. She died of peritoneal metastases 3 months postoperatively.
10	Miyamoto et al. Japan 1983 [22]	F	61	Adenocarcinoma and squamous cell carcinoma with malignant osteoid and cartilage.	Not done	Not done	Patient died 3 months after surgery.
11	Born et al. USA 1984 [23]	F	90	Malignant glandular structures in multilayered fashion. Malignant cartilaginous tissue.	Not done	Not done	Tumor was invading duodenum. Patient died on the third postoperative month from intractable pulmonary sepsis. Permission for autopsy was not granted.
12	Inoshita et al. Japan 1986 [24]	M	53	Adenocarcinoma, papillary and cribriform pattern. Sarcomatous component with interlacing bundles of highly atypical spindle cells admixed with malignant cartilage, osteoid, and foci of rhabdomyoblastic cells.	Positive for EMA	Rhabdomyoblast cells were positive for myoglobin	Died 17 months postoperative resection. Metastases to liver (Carcinomatous and RMS), and to the pancreas, diaphragm, and regional lymph nodes (RMS component only)
13	Uesaka et al Japan 1995 [25]	F	54	Carcinomatous glandular and ductal structures, nests of squamous cell carcinoma. Sarcomatous component with non-calcified osteoid differentiation and a small island of malignant cartilaginous tissue.	Positive for CEA, EMA, and Cytokeratin	Positive for EMA, CK, and S-100 protein in chondroid foci. Negative for vimentin and CEA	Portal tumor thrombus of sarcomatous elements, and osteoid differentiation. Metastatic adenocarcinoma to one lymph node. Patient died of liver metastases 3 months after surgery.
14	Yavuz et al. Turkey 2000 [26]	F	50	Adenocarcinoma with tubular and cribriform structures. Sarcomatous component shows interlacing bundles of spindle cells with small islands of malignant cartilage and many rhabdomyoblasts.	Positive for cytokeratin and EMA	Diffusely positive for vimentin. Rhabdomyoblasts positive for desmin.	Patient died three months after diagnosis due to disseminated spread of the tumor.
15	Hotta et al Japan 2002 [13]	F	53	Carcinomatous component with tubular formations. Spindly and pleomorphic sarcomatous component with few foci of chondroid formation	Positive for cytokeratin, EMA and CEA	Positive for vimentin. Some tumor cells positive for cytokeratin	Patient underwent three surgical procedures. Developed leakage at anastomosis site and severe infection. Developed many liver metastases, and died of liver failure on 61st post-operative day.
16	Ajiki et al. Japan 2002 [27]	F	69	Well and poorly differentiated tubular adenocarcinoma. Sarcomatoid tissue with chondroid differentiation.	Positive for Keratin	Focally positive vimentin and cytokeratin. Positive for S-100 protein	Metastatic sarcoma with chondroid differentiation in the lesser omentum. The tumor had spread to the liver. The left kidney showed a mass (clear cell carcinoma). He died 7 months later with peritoneal dissemination.
17	Sakurai et al. Japan 2006 [28]	F	54	Poorly differentiated tubular adenocarcinoma and spindle-cell sarcoma with differentiation to cartilage and partly bone	Not detailed	Positive cytokeratin staining in malignant cartilage..	Tumor was advanced involving the transverse colon and the duodenum. Patient received chemotherapy. He developed liver metastases and died 15 months following surgery.
18	Okamura et al. Japan 2006 [29]	F	60	Adenocarcinoma Sarcomatous tumor cells with a spindle shape and partial chondroid differentiation.	Positive for epithelial markers.	Chondroid areas are positive for S100 protein.	Patient had long survival and was alive 54 months following surgical resection.
19	Oberoi et al. India 2006 [30]	M	68	Well differentiated adenocarcinoma and poorly differentiated sarcoma with chondroid areas.	Positive for cytokeratin	Positive for vimentin and cytokeratin	Direct invasion of the liver and metastatic deposits to the omentum, mostly adenocarcinomatous component. No data on outcome.
20	Zhang et al. USA 2008 [4] 4	M	65	Squamous cell carcinoma, with mesenchymal spindle cell component and malignant chondroid elements.	Not available.	Not available.	Alive at three months following surgery.
21	Kohtani et al. Japan 2009 [31]	M	84	Adenocarcinoma admixed with malignant mesenchymal elements including bone and cartilage.	Positive for cytokeratin and EMA but negative for CD68 and desmin	Positive for CD68 but negative for cytokeratin and EMA	Cholecystectomy only was done, followed by low dose chemotherapy. Patient survived four years postoperatively.
22	Krishnamurthy et al. India 2011 [32]	M	83	Undifferentiated carcinoma spindle and giant cell type with osteoclastic giant cells. Focal chondrosarcoma.	Positive for Cytokeratin and CD68 (in osteoclastic giant cells)	Not documented	Gallbladder was friable necrotic and removed piecemeal. Died on the 10^th^ postoperative day of atrial fibrillation and cardiogenic shock.
23	Coetzee et al., South Africa 2011 [33]	F	38	Malignant glands with serrated architecture, focal adenoca in-situ. Pleomorphic and atypical stromal cells showed a myxoid background with chondroid appearance, focal osteoid was present.	Positive pancytokeratin AE1/3 in dysplastic lining epithelium	FNA specimen: Positive MSA in stromal cells.	Patient had cholecystectomy. He returned nine months later with dyspnea and jaundice. Ultrasound-guided FNA of mass under diaphragm: biphasic tumor, stromal component predominated.
24	Parreira et. al., Brazil 2012 [34]	F	58	Well-differentiated adenocarcinoma, papillary and tubular, with foci of squamous cell carcinoma. Chondrosarcomatous, osteoid and atypical tissues.	Not done	Not done	Patient died six months following surgery due to multiple metastases.
25	Kim et al., South Korea 2012 Case 1 [35]	F	72	Foci of malignant glands. Widespread crossing bundles of mesenchymal components with chondroid differentiation.	Positive for: Cytokeratin, CK20, CK7, CEAE-Cadherin, Ki67 and p53	Positive for vimentin, osteocalcin, S-100 protein, Ki67 and p53 and SMA. Negative for myoglobin, and desmin.	Tumor progressed with direct invasion of the colonic loop, stomach, and liver.
26	Sadamori et al. Japan 2012 [36]	M	80	Moderately to poorly differentiated adenocarcinoma. Sarcoma with osteosarcomatous and chondrosarcomatous elements.	Not done	Not done	Died due to multiple liver metastases 13 months after surgery.
27	Aleaga et al. Cuba 2013 [37]	F	72	Moderately differentiated adenocarcinoma. Sarcomatoid stroma with pleomorphic cellular areas and malignant chondroid matrix.	Positive CAM 5.2, focally positive for CEA.	Positive for vimentin and S-100 protein.	Patient died 7 months following surgery with multiple liver metastases.
28	Kishino et al. Japan 2014 [38]	F	70	Adenocarcinoma. Sarcomatous element of malignant spindle cells, Foci of malignant cartilage and bone.	Positive for cytokeratin (AE1/AE3).	Positive for vimentin, myogenin and SMA.	Liver metastasis developed 19 months after cholecystectomy, and partial hepatectomy was performed. No tumor recurrence after two years following operation.
29	Faujdar et al. India 2015 [39]	F	60	Carcinomatous element with moderate anaplasia. Sarcomatous elements in the form of spindle cells arranged in bundles and fascicles, and heterologous elements of cartilage and mature bone.	Positive for cytokeratin.	Positive for vimentin	Not documented. Patient was lost for follow up.
30	Al Ratroot et al. Saudi Arabia 2018 (Current Case)	F	52	Solid malignant epithelial nests within highly malignant sarcomatous stroma with scattered islands of malignant cartilage. Intracytoplasmic eosinophilic hyaline globules present.	Positive for cytokeratin. Negative for vimentin.	Positive for vimentin and desmin. Cartilaginous islands positive for S100 protein.	Metastases to the liver, omentum, peri-colic and peri-gastric fatty tissue and lymph node. Patient lost for follow up after seven months.
